# Self-Concepts in Reading and Spelling among Mono- and Multilingual Children: Extending the Bilingual Advantage

**DOI:** 10.3390/bs9040039

**Published:** 2019-04-13

**Authors:** Julia Festman, John W. Schwieter

**Affiliations:** 1 Department of Primary and Secondary Education, Pedagogical University Tyrol, 6020 Innsbruck, Austria; 2 Diversity and Inclusion Research Group, University of Potsdam, 14469 Potsdam, Germany; 3 Multilingualism Research Team, Center for Didactics, Pedagogical University Tyrol, 6020 Innsbruck, Austria; 4 Language Acquisition, Multilingualism and Cognition Lab, Wilfrid Laurier University, Waterloo, ON N2L 3C5, Canada; jschwieter@wlu.ca

**Keywords:** domain-specific self-concept, academic achievement, metacognition, executive functions, multilingual children, reading comprehension, reading fluency, spelling

## Abstract

Cognitive representations and beliefs are what comprise an individual’s self-concept. A positive self-concept is related to and influences academic achievement, and the relationship between a domain-specific self-concept and achievement in the same domain is positive and strong. However, insufficient attention has been paid to these issues among multilingual children. More importantly, since instruction strongly contributes to the development of metacognition and executive functions (EFs), and since the bilingual advantage hypothesis holds that the constant management of multiple languages entails benefits for EF, we bring together these important issues in the present study. We examine the relationship between domain-specific self-concepts and standardized assessment of reading and spelling competences against the background of potential differences in self-concept between monolingual and multilingual German children. While between-group comparisons revealed no significant differences for self-concept nor reading competency, monolinguals outperformed multilinguals in spelling. Correlations between domain-specific self-concepts and academic achievement in reading comprehension, reading fluency, and spelling were positive and significant for both groups. Regardless of language background, children’s evaluations of their academic achievement (reading and spelling) were realistic. We argue, on a theoretical basis, that metacognition and EFs could facilitate a bilingual advantage and improve educational outcomes.

## 1. Introduction

The notion of self-concept comprises individuals’ cognitive representations and beliefs of themselves [1]. An overall positive self-concept has been shown to have a positive influence on academic achievement [2] and the relationship between domain-specific self-concepts and achievement in that same domain is positive and strong [3]. The body of research reporting on these issues among multilinguals is scant even though it has been shown that instruction strongly contributes to the development of metacognition and executive functions (EFs) [4]. This gap is even more surprising since ongoing work has documented the possible impact of multilingualism on cognition (the so-called bilingual advantage), i.e., the constant management of multiple languages may consequentially benefit executive functions [5]. EFs are comprised of “a variety of self-regulatory processes including goal-directed intentional behavior, cognitive processes that allow flexibility, error detection, and conflict resolution” [6] (p. 152). In the present study, we bring these important issues together by analyzing self-concepts of reading and spelling among third-grade monolingual and bilingual children in Germany. In the next sections, we provide a background of the notion of self-concept followed by a discussion of recent work that has examined EF, metacognition, and academic achievement. On this backdrop, we present the current study’s research questions, hypotheses, and findings. In doing so, we draw on the ongoing ‘bilingual advantage debate’ to hypothesize how these issues may develop distinctly among monolingual and bilingual children and how future work may examine them. Finally, we discuss the implications of the results and offer suggestions for practice and for future research trajectories.

## 2. Development of Self-Perceptions and Self-Beliefs to Self-Concept

According to Harter and Pike [7], self-concept genesis commences during early childhood. It is predominantly characterized by children’s exaggerated positive self-beliefs formed through experience with the environment, and is progressively differentiated with increasing age [8,9]. Environmental reinforcements and significant others seem to play a key role in refinement of self-beliefs [1]. For instance, upon entering school, children become aware of external evaluations concerning their personalities in terms of convictions and judgements stated by parents, relatives, teachers, and classmates. By integrating these into the child’s already existent self-perception, his/her beliefs of self-competencies may change [8,10,11]. During the third grade, self-perceptions develop into realistic evaluations of one’s self [12,13]. Children’s emerging self-concept is considered to be a complex, multifaceted and multidimensional construct consisting of different domains [9,14,15]. It is comprised of a global, general dimension as well as nonacademic (social, emotional, and physical self-concepts) and academic ones predominantly related to different school subjects [1].

### 2.1. Academic Self-Concept

The importance of a positive self-concept for healthy identity and personality development [16] has advanced research on self-concepts in particular in the educational fields [17] (for a review, see [18]). In the field of educational psychology [19], special emphasis lies on the domain-specific academic self-concept conveying students’ perceptions and beliefs about their academic abilities, competences, and performance. The academic self-concept often is divided into two independent self-concepts: verbal and mathematical. The verbal self-concept incorporates all aspects pertaining to native and foreign languages as well as societal fields; in contrast, the mathematical self-concept refers to natural sciences [9,10,19].

### 2.2. Causal Ordering of Academic Self-Concept and Academic Performance

Based on earlier research, the causal ordering of self-concept and academic achievement was largely unclarified as two contrasting models put forward by Calsyn and Kenny [20] dominated self-concept research [19,21]. According to one of these, the self-enhancement model, academic self-concept is predictive of later academic achievement. Support for this model can be found in a comprehensive meta-analysis of longitudinal studies concerning self-concepts and academic performance [18]. In contrast, the skill development model assumes that self-concept emerges from prior academic achievement [20,22,23].

More recently, it has been suggested that self-concepts and academic achievement act reciprocally with one another as put forward in the reciprocal effects model [19,24,25]. This model assumes that “positive self-concept enhances achievement and higher achievement fosters self-concept” [17] (p. 277). The vital role of positive self-beliefs being a critical determinant not only for academic performance, but also for the healthy development of the individual, contributes to the proclamation of seeing self-concept enrichment as “a central goal of education and an important vehicle for addressing social inequities experienced by disadvantaged groups” [19] (p. 60).

### 2.3. Domain-specific Self-Concept and Academic Self-Concept of Disadvantaged Children

Previous research provides evidence for a close linkage between domain-specific academic self-concept and academic achievement in the corresponding domains [2,13,18,26]. The significant correlations between academic self-concepts and respective academic performance are not dependent upon gender and they seem to intensify with age according to growing competences and experiences throughout the school year [18].

The relationship between academic achievement and academic self-concept among disadvantaged groups such as immigrant children, however, is unclear. Niehaus and Adelson [17] compared self-concept comprising domains of reading and mathematics among native English-speaking children and English language learners in the US. The children’s grade level ranged from the end of kindergarten to third-grade and they had either Spanish or an Asian language as their first language (L1). The results revealed significant differences in academic domains of self-concept between the groups; whereas children with L1 Spanish scored higher in self-concepts for reading, mathematics, and all school subjects compared to native English speakers, children with Asian language backgrounds reported higher self-concept levels only for mathematics. 

Eccleston, Smyth, and Lopoo [27] examined academic self-concept, general self-esteem, and academic achievement in a large sample of 10–19-year-old African-Americans and European-Americans in schools. The results suggested differences between the two groups such that African-American students showed higher levels of self-esteem and academic self-concept (e.g., in reading) but displayed lower scores in academic achievement (e.g., reading). This was not the case for the European-American students. The authors explained this apparent unrealistic self-perception by suggesting that African-American students were more likely to discount negative feedback from their teachers than European-American peers in a way that does not influence their self-concept. It should be noted, however, that academic achievement was only based on teachers’ evaluation of the students’ academic abilities, and that this data was only available for a very small subset of the students from the sample.

Other studies have also revealed inconsistencies between self-concept and actual achievement. For instance, Mücke [28] investigated general academic self-concept and academic performance in reading, reading comprehension, and spelling among first- and second-grade immigrant and nonimmigrant children in Germany. Interestingly, while there was a significant correlation between self-concept and academic achievement for nonimmigrant children, this was not the case for immigrant children. In line with the observations made for the African-American students from Eccleston et al.’s [27] study, these results seem to indicate that immigrant children’s estimate of their academic competence was less realistic than that of their nonimmigrant peers.

## 3. EF, Academic Achievement, Self-Concept, and the Bilingual Advantage Debate

Previous work has found a positive correlation between EF and academic achievement among children (for a review, see Ref [29]). Bull and Scerif’s [30] study reported a significant relationship between behavioral measures of attention shifting, working memory, and inhibition- and performance-based math measures. Similar results were found in a later study by Latzman, Elkovitch, Young, Anna, and Clark [31] in which the researchers reported a positive association between inhibitory abilities and achievement in mathematics. The study also found that cognitive flexibility and monitoring had a positive correlation with reading performance. 

It is plausible that there is an association between EF and self-concept; however, research testing this has been scant. Roebers et al. [6] was perhaps the first of only two studies to our knowledge that has explored the associations between EF, metacognition, and self-perceived competence in the context of early academic outcomes. In this longitudinal study, the researchers examined the effects of performance-based EF and academic self-concept on academic achievement among 209 first graders (mean age 7;6). One year later, the same children’s EF and academic self-concept were once again measured along with their metacognitive control and monitoring and their math and literacy skills. The results suggested that EF was significantly related to metacognitive control and that self-concept was significantly associated with metacognitive monitoring. These findings were both significant cross-sectionally and longitudinally. There were differential effects on academic outcomes, such that EF was associated with literacy and math, whereas metacognitive control was related only to literacy. Roebers et al. [6] argued that the predictive power of EF for academic achievement is more general, whereas the effects of metacognition are more restrictive. 

A subsequent study by Spiess, Meier, and Roebers [32] showed a concurrent relation between EF and metacognition among second graders. However, while these two constructs at the beginning of the school year were significantly related, by the end of the year, it was apparent that earlier EF or metacognition was not predictive of subsequent EF or metacognition. This suggests that the development of EF and metacognition do not entirely depend on one another but instead follow distinct paths.

Because EF and metacognition are higher-order cognitive processes that develop through childhood as a result from active and continuous interaction in a natural environment, Roebers [4] advocates for a unified framework of cognitive self-regulation, which integrates EF and metacognition and explains the emergence and development of cognitive self-regulation. At the core of this discussion is the dynamic relationship between EF and metacognition during child development. Roebers [4] notes that “the relationship between EF and metacognition within a broader conceptualization of cognitive self-regulation is likely to change over time, as [the two] undergo substantial improvements in childhood” (p. 46). However, Roebers [4] further states that more sophisticated skills develop “only if an individual receives direct instructions, close supervision in critical situations or challenging tasks, and feedback from skilled partners. Such factors allow the child to experience the benefits and possible use of EF or metacognition” (p. 41). 

When considering recent claims that bilinguals may have heightened EF compared to their monolingual counterparts due to the simultaneous development and continuous use of two language systems (for reviews, see Ref [5,33], but see Ref [34] for alternate explanations), it is possible that bilingual children may also have more abundant and richer opportunities to experience the benefits and possible use of EF and metacognition, which in turn could have consequences for their development of self-concept. A number of studies using a variety of measures (e.g., the Simon task, the attention network test, and the dimensional change card sort task) have found enhanced EF in bilingual children compared to monolingual children [35,36,37,38,39,40,41]. These studies suggest that bilingual children have better EF due to “the demands placed by bilingualism on brain networks and structures within them that subserve domain general EFs” [33] (pp. 398–399). Our study is the first to our knowledge that will begin to look at whether these issues will have consequences for self-concept. It also offers a new area of inquiry within the bilingual advantage debate which has the potential to inform not only the relationship between self-concept and EF for both monolinguals and multilinguals, but also the pedagogical interventions that may facilitate these effects. In the next sections, we present the current study, the findings, and implications for ongoing and future research. 

## 4. Present Study

### 4.1. Motivation of the Study

Prior research has scarcely related specific academic self-concepts to academic achievement, and none has done so within the context of the bilingual advantage debate. Mücke [28] associated general academic self-concept with domain-specific academic achievement in terms of basal reading, reading comprehension, and spelling competences of first- and second-grade children. Conversely, a closer examination of domain-specific academic self-concepts and corresponding domain-specific academic performance remains unconsidered but might provide the best approach to shed light on the specific interplay between self-concept and academic achievement. It should also be noted that in former studies [42], academic achievement has often been determined by teachers’ or students’ self-reported grade-point average. Given this potential threat to reliability, we must interpret these evaluative methods with caution and complement or replace them with standardized assessments.

Studies on academic self-concepts and their impact on academic achievement have predominantly focused on specific ethnicities of immigrants and their contrasting juxtaposition to nonimmigrant students. There is comparatively little self-concept research examining the diversity of children’s language backgrounds—an issue that dominates today’s classrooms across the globe. Along with massive waves of migration comes an increase in multilingualism. However, multilingualism is not necessarily equivalent to migration background [43], even though the latter is commonly referred to in former studies, and in Germany, lower socioeconomic status is often highly confounded with migration status [44]. Ideally, studies should include highly-diverse samples in terms of language backgrounds rather than focusing on a certain ethnicity of immigrants. For instance, in Germany, there has been only one study to our knowledge [28] that has explored the conjunction between self-concepts and academic performance of first- and second-grade immigrant and nonimmigrant children. Investigations involving third-grade primary school children have focused less on formal academic self-concept research as many studies included a larger age range. However, the fact that academic self-concept is considered to be largely consolidated at that age [12,13] suggests that it might be a particularly interesting age to scrutinize the interplay between domain-specific self-concept and actual academic achievement. 

### 4.2. Research Questions and Predictions

In the present study, we investigated a heterogeneous group of third-grade mono- and multilingual children in primary schools in Germany. The children were grouped according to the number of languages spoken in their household: either monolingual or bi-/multilinguals. Importantly, the multilingual group was composed of children from diverse language backgrounds while earlier studies were based on single-ethnicity background samples.

In order to explore the potential group differences of domain-specific self-concept, we first examined verbal academic self-concept, more specifically reading and spelling, given that they display vital competences for academic success [12,45,46] and are key objectives in primary school education. We predict that the multilingual group would have more positive self-concepts compared to the monolingual group.

Secondly, we assessed the two groups’ academic achievement in reading fluency, reading comprehension, and spelling to determine whether there were differences between the monolingual and multilingual children. We used standardized tests rather than self-reported grade point averages to ensure reliability of our analyses. In prior work, we have shown that due to smaller lexicon size in German, multilingual children fair poorer in spelling compared to age-matched monolinguals [47]. We predict that with equal reading support provided in our inclusive schools, children’s reading fluency and reading comprehension should not differ between groups. 

Thirdly, as in Mücke [28], we used a correlational analysis to examine the informative value of children’s self-concepts pertaining to their actual performance in reading fluency, reading comprehension, and spelling. However, in the present study, we specifically focus on their domain-specific self-concepts. We hypothesize that adequate external evaluations of academic achievement provided by the teachers in the three inclusive schools, supported the development of a differentiated self-concept such that the pupils could pass realistic evaluations of their academic competence.

### 4.3. Participants

The participants included 125 third-grade children who were enrolled in three primary schools at different locations in the greater Berlin area. These inclusive schools were characterized by a clear positive acknowledgement towards diversity. The initial sample was larger (N = 168), but, due to incomplete questionnaires, incompatibility of child and parent answers concerning language background information or reported language contact at locations other than their homes, 43 children were excluded prior to the analyses. The participating children were classified as monolinguals (N = 69; 33 female, mean age 107 months) and multilinguals (N = 56; 30 female, mean age 111 months). This group classification was done according to L1 backgrounds other than German that was reported in a self-designed questionnaire which we describe in the materials section (please refer to 4.4.1). Accordingly, for monolingual children, German was the only language spoken at home exclusive of any further language contact. Those categorized as being multilingual included bilingual (N = 50; 24 female) and trilingual children (N = 7; 3 female). Multilinguals spoke at least one other language at home in addition to German, denoting the child’s consistent use of L1 with an adequate level of respective verbal proficiency. There were no differences in their parents’ aspiration regarding their children’s German competences nor the children’s evaluation of their teachers’ behavior towards them (see Table 1).

The multilinguals in our highly-diverse sample spoke 20 different languages, including Albanian, Arabic, Bosnian, Chinese, Edo, English, Greek, Hebrew, Hungarian, Kurdish, Mandinka, Persian, Polish, Punjabi, Rumanian, Russian, Serbian, Spanish, Turkish, and Zaza. Overall, 83% of multilingual and 7% of monolingual children had life experience with migration when considering their own or their parent’s place of birth as the decisive criterion (at least one parent had to be born outside of Germany).

Consistent with the Declaration of Helsinki, the approval of administration was granted from the head of the schools, the Ministry of Education, Youth and Health (Land Brandenburg), and the Senate for Education, Youth and Science (Berlin). All parents gave their informed consent for their inclusion in the study and their children’s before participating. Ethical clearance was obtained from the ethics committee of the University of Potsdam (11/2015). 

### 4.4. Materials

#### 4.4.1. Descriptive Background Information

The descriptive background information was assessed by means of a self-designed child- and parent questionnaire. Note that we report only the information relevant for this paper, i.e., both children and parents were asked to provide the child’s gender and age. Furthermore, children and parents were asked to specify their family’s home language (to group the children as mono- or bi-/multilingual) and the country of birth of child and parents (to determine the child’s migration status).

To gather information on the parents’ educational aspirations for their children, we asked the parents about the importance of their child’s correct spelling and their effort made in German lessons (Table 1, upper part). In the children’s questionnaire, we asked the children to evaluate their teachers’ behavior towards them across different aspects (e.g., fair treatment, scolding, friendly communication, and care taking) (refer back to the lower portion of Table 1). 

#### 4.4.2. Domain-Specific Self-Concepts

We used two scales in the above-mentioned child questionnaire to assess the domain-specific self-concepts. The children were asked to evaluate their reading and spelling competences according to a respective five-item scale inspired by the FEESS 3–4 (“Fragebogen zur Erfassung emotionaler und sozialer Schulerfahrungen” [Questionnaire to assess emotional and social education experiences] [48]). The questions were constructed in a way such that children were more likely to comprehend valid information [49].

The reading self-concept scale comprises the items “Ich kann gut lesen.” [I’m good at reading], “Ich kann auch schwierige Texte verstehen.” [I can even understand difficult texts], “Im Lesen schaffe ich nur einen Teil der Aufgaben.” [I can only manage to do part of the reading tasks], “Beim Lesen verstehe ich viele Wörter.” [When reading, I understand many words], as well as “Beim Lesen verstehe ich nur sehr wenige Wörter.” [When reading, I understand only a few words]. Each item is scored on a four-point scale, where a score of 1 would designate little competence and a score of 4 would be the highest level of competency (minimum test value of 5, maximum value of 20 for the entire scale). Internal consistency of the total scale is α = 0.81 (Cronbach’s alpha). 

The spelling self-concept-scale equally covers five items including “Ich bin gut im Rechtschreiben.” [I’m good at spelling.], “Ich muss viel üben, um im Rechtschreiben gut zu sein.” [I have to practice a lot to be good at spelling.], “Im Rechtschreiben schaffe ich nur einen Teil der Aufgaben.”, [I can only manage to do part of the spelling tasks.], “Im Rechtschreiben verstehe ich die Regeln, die der Lehrer erklärt.”, [I understand the spelling rules explained by the teacher.], “Im Rechtschreiben verstehe ich nur sehr wenig.” [I know little about spelling.]. Scoring and associated values are equivalent to the scale determining reading self-concept. Internal consistency of the spelling scale is α = 0.68 (Cronbach’s alpha). 

#### 4.4.3. Standardized Tests of Reading and Spelling

Reading comprehension was assessed with a paper–pencil subtest of the standardized reading test ELFE 1–6 (“Leseverständnis-Tests für Erst- bis Sechstklässler” [Reading comprehension test for grades 1–6], test booklet A [50]). The subtest determining written word-recognition consisted of 72 items of 1–4 syllable words. The children were given three minutes to look at the picture and then to select and underline the printed word (out of four) that matched the picture. From among the possible response options, three of them were distractors such that they were phonologically or semantically similar to the target item. The total scores of correct responses could range from 0 to 72 points (1 point for each correct answer). The test handbook reports high internal consistency of α = 0.96 (Cronbach’s alpha) for third-grade children [50].

To test reading fluency, we used the standardized reading word fluency test SLRT-II (“Salzburger Lese- und Rechtschreibtest” [Salzburger reading and spelling test], [51], form A). In individual sessions, children were presented with a list of 156 words that increased in complexity. They were given one minute to read aloud as many items as possible. Responses were tape recorded and documented by the researcher. The reading fluency score reflects the number of items read correctly per minute. The reported inter-method reliability is r = 0.94 for the word list [51].

Orthographic spelling competences were measured by the subtest of orthographic competence (list 3), taken from BUEGA (“Basisdiagnostik Umschriebener Entwicklungsstörungen im Grundschulalter” [Basic diagnostics of circumscribed developmental disorders of primary school age children]) by Esser, Wyschkon, and Ballaschk [52]. Stimulus items (17 different words increasing in complexity) were prerecorded and played one-by-one to the children. After the practice items, we asked the children to successively write down the words on a tablet computer (Microsoft Surface Pro 2 tablet, display size: 25.5 cm × 17 cm, resolution: 2160 pixels × 1440 pixels). The spelling score was given based upon the number of words spelled incorrectly. The maximum number of errors could be 17 (all words spelled incorrectly), and the best possible score could be 0 (all words spelled correctly). The test handbook reports an internal consistency of α = 0.83 (Cronbach’s alpha) for the target group of nine-year-old children [52].

### 4.5. Procedure and Data Analyses

This study is part of a larger project. Hence, we report only the tasks relevant for this study. The parents completed the parents’ questionnaire at home. The version of the questionnaire for the children was used in a group session taking 45 min with one experimenter reading and explaining each question before the children wrote down their answers. That way we ensured that lack of reading abilities did not interfere with filling-in the questionnaire. Both child and parent questionnaires were administered in paper–pencil format. Furthermore, reading comprehension and orthographic competences were assessed, the latter on a tablet computer. To be able to record children’s individual verbal responses, we administered the test for reading fluency in an individual session.

All statistical procedures were carried out with SPSS version 24 (IBM Corp., Armonk, NY, USA). The normality of the distributions was checked with the Shapiro–Wilks test and all parameters were non-normally distributed. We conducted a Mann–Whitney test to determine whether there was a significant difference between the groups regarding background information (e.g., age, gender, and teachers’ behavior), in domain-specific self-concepts (reading and spelling), and in performance on the standardized tests (spelling, reading fluency, and reading comprehension) (outcome values: *U*- and *p*-values). We considered results of *p* < 0.05 to be significant. After the data was converted to ranks, we ran correlations with Spearman’s rho as a nonlinear rank correlation coefficient to investigate whether children’s domain-specific self-concepts and their actual academic achievements correlated.

## 5. Results

### 5.1. Descriptive Statistics and Group Comparisons

On average, both groups scored on the upper end of the four-point scale, indicating positive self-concepts in both assessed domains: for reading (mean mono 3.27, SD 0.69; mean multi 3.19, SD 0.61) and for spelling (mean mono 3.18, SD 0.69; mean multi 3.02, SD 0.57). Table 2 reports the mean rank of the self-ratings of domain specific self-concepts for both groups for reading and spelling. Mann–Whitney tests revealed that there was no significant difference between both groups in their domain-specific self-concepts.

The results of the standardized tests are presented in Table 3. Mann–Whitney tests show that for reading comprehension and reading fluency, the performance of both groups did not differ significantly. However, performance on the standardized spelling test (BUEGA) was significantly different between the groups with monolinguals producing fewer errors than multilinguals. 

### 5.2. Correlations

For reading, correlations between the rating for domain-specific self-concept and the performance scores on the two different standardized tests are presented separately for the monolingual and the multilingual group in Table 4. The correlation between reading comprehension abilities and the self-concept for reading, although weak, was significant for both groups. In both groups, reading fluency was significantly correlated with the assessed reading fluency and had a moderate effect size.

For spelling, correlations between the rating for domain-specific self-concept and the performance score on the standardized test (BUEGA) are presented separately for the monolingual and the multilingual group in Table 5. For both groups, there was a significant correlation between self-concept for spelling and their performance on the spelling test. The effect size for this correlation was moderate. 

## 6. Discussion

In this study, we investigated domain-specific self-concepts in the verbal domain and academic achievement, with a particular focus on reading and spelling as they display fundamental aspects of academic achievement in general [12,45,46]. Our sample consisted of two groups of third-graders who were categorized as mono- or multilingual according to their language background. We investigated whether there are between-group differences in domain-specific self-concepts and associated academic performance. We subsequently correlated domain-specific reading and spelling self-concepts and associated academic performance for each group separately to reveal possible group-related differences in self-evaluation ability and reference to reality (i.e., academic achievement). 

The findings of our study suggest no group-related differences in self-concept of reading and spelling. This is a remarkable finding in contrast to other studies [17,27,42]. Regarding differences in performance on standardized tests of academic achievement, there were no group differences in reading (comprehension and fluency), but there were in spelling with lower performance of the multilingual group. Overall, we observed a realistic evaluation of academic achievement for both groups. Contrary to other studies (e.g., Ref [28]), our study showed no discrepancies between the two groups in the third-graders’ ability to realistically evaluate their reading and spelling. 

### 6.1. Domain-Specific Self-Concepts

The monolingual and multilingual children in our study have comparatively high domain-specific academic self-concepts. This is consistent with findings from previous studies showing that children in third grade often have high levels of self-concept compared to children at the very end of primary school [11,53]. Because a child’s self-concept is considered to consist of interpretations of past experiences [54], these results furthermore suggest that feedback processes of teachers and parents as well as comparisons among the children themselves within educational contexts (which are essential for a healthy self-concept development) equally have an effect for both groups regardless of the children’s language background. 

Previous studies reported inconsistent differences in self-concept evaluations between immigrant and nonimmigrant groups (e.g., Ref [17,27,28,55]). Contrarily, the results of our study indicate no significant differences in domain-specific verbal self-concepts between mono- and multilingual children, for reading and spelling. This is a gratifying finding as it may imply that efficient and adequate feedback processes and support of parents and teachers (please refer back to Table 1) mainly contributed to the development of children’s adequate and healthy self-perception. As we could show by including the questionnaire responses especially provided by the children in our study, their evaluations of their teachers’ behavior towards them (in terms of fairness, friendly way of communication, caring, etc.) was equally perceived by the children in both groups, characterizing the supportive school environment. The fact that there were no respective differences in self-concept between mono- and multilingual children in this sample may even suggest a shift in diversity management: teachers and other educational staff might be better able to deal with heterogeneity in their classrooms. They seem to give equal treatment and offer equal opportunities to all children including attention to special needs and different demands that most notably need to be taken into account in heterogeneous learning settings such as our selected schools [56]. Accordingly, we might assume that greater awareness and conscious handling of diversity in the classroom [56] (possibly implemented by educators at our selected schools) was able to meet the diverse needs of children—and no longer has to inevitably lead to disadvantages for multilingual children as postulated earlier by Marsh and Martin [19].

### 6.2. Academic Achievement

In our study, there were no significant differences between mono- and multilingual children with respect to their performance in reading comprehension and reading fluency. However, monolinguals outperformed their multilingual peers in spelling [47]. Pertaining to academic achievement in general, the majority of studies examining academic performance of mono- and multilingual children suggest that multilingual children display lower academic performance compared to their monolingual peers (e.g., Ref [27,28,42,57]). With regard to specific competences of reading and spelling, previous research results are conflictive.

For reading comprehension, the findings are in line with results revealed by Verhoeven [58] who did not find significant differences between mixed L1-language speakers with Dutch as a second language (L2) and native Dutch children at the study onset. Some studies provided evidence for L2 learners performing at the same level as monolinguals [59,60], whereas others reported inferior reading comprehension skills for L2-learners (e.g., Ref [61]; for a comprehensive review on reading comprehension, see Ref [62]).

For reading fluency, our selected sample is reminiscent of findings made by da Fontoura and Siegel [63], namely that mono-and multilingual children do not differ in their reading fluency. These results suggest that both groups of children might be equally familiar with written discourse presumably reflecting, among other things, respective aspirations and encouragement of those involved in promoting mono- and multilinguals’ reading competences in today’s classroom (refer to Table 1). Consistent with observations made by da Fontoura and Siegel, multilingualism must not necessarily be associated with reading difficulties. In line with Verhoeven [58], monolinguals in our study outperformed multilingual third-graders in spelling. In accordance with Verhoeven’s conclusions, it could be assumed that, for multilingual children learning Dutch, the phoneme-to-grapheme conversion seems to be more challenging than the reverse process due to multilinguals’ lower proficiency concerning phoneme distribution in the required language and the associated matching of phonemes with corresponding orthographic patterns [58]. With respect to the present study, these assumptions can at least be validated for German as the target language. As orthographies differ across languages concerning their complexity and transparency, broad generalizations to other languages should be treated with caution. For the parents in our study, it was equally important that their children—irrespective of language background—learn how to write texts without errors. There was no correlation between parents’ judgement of the importance of this skill with the spelling test performance of their children (*r_s_* = −0.128; *p* = 0.171).

### 6.3. Juxtaposing Constructs: Domain-Specific Self-Concepts and Academic Achievement

In our study we closely examined particular aspects of academic achievement (reading and spelling) and found similarities between mono- and multilingual children: Reading self-concept and actual reading competences (i.e., reading comprehension and fluency) were essentially the same for both groups. Analogous conjunctions could be determined for spelling self-concepts and associated spelling performance of both groups. They realistically evaluated their academic performance in terms of reading and spelling regardless of their language backgrounds and had the same positive evaluation of their teachers’ behavior towards them and support for them.

This is in contrast to Mücke’s [28] study in which there was no correlation between self-concept and academic performance for immigrant children. On the one hand, the more realistic judgment of reading and spelling abilities in multilingual children in our study could be explained by the fact that Mücke only used a general academic self-concept, whereas we asked about the specific self-concept of reading and spelling. Hence, our study fills a gap in the ongoing work being done in this area. In direct comparison with monolingual third-graders, the multilinguals in our study provided equally profound and differentiated judgements of their academic performance; they were able to go beyond merely evaluating quantitative aspects of respective competences (e.g., reading fluency). Multilingual children were equally capable of determining qualitative aspects of their academic performance (e.g., reading comprehension and correct spelling). 

### 6.4. EF, Self-Concept, and a Bilingual Advantage

On the other hand, an alternative explanation in our study is possible for the multilingual children’s capability to pass realistic judgments of their own spelling and reading skills. It is plausible that the multilingual children in our study were able to make efficient use of the supportive learning environment which guided them equally well through the challenges of going to school, learning to read and write, learning to control their actions, and improving their self-regulation. The specific ways in which they were capable of doing this are still unknown and merits further investigation. However, in accordance with Roebers’ [4] broader idea of cognitive self-regulation (including EF and metacognition), it is quite possible that for the multilingual participants in our study, the development of a differentiated and realistic domain-specific self-concept is promoted by their benefits of EF and metacognition.

Being a multilingual, growing up with different languages, having experiences with migration, integration, and inclusion, are all factors that make it a “challenging task” to identify what exactly triggers the development of more sophisticated skills [6]. As was seen in the children’s perception of their teachers’ behavior in class towards them (refer back to Table 1), there was no difference between the mono- and the multilingual children. The feeling of being treated in a fair manner, being cared for, etc. was essentially the same for the groups. This draws a picture of the teacher-pupil relationship as one being characterized by acceptance, friendliness, fairness, supportive communication, along with kind, helpful feedback. We believe that this supportive learning environment helped the children in both groups to develop a realistic self-concept for specific skills, namely reading and spelling. The teachers in these schools seemed to have managed to create learning environments in which children did not have to discount negative feedback in order to create their self-concepts for school-related skills (see Ref [27] for an explanation of the discrepancy between self-concept and academic achievement among African-American students). This might also explain the difference in findings from our study and Mücke’s [28]. Therefore, we argue that if multilingual children can focus on training their academic skills, rather than having to face negative feedback in their learning environment, their possibly enhanced EFs might promote a differentiated development of a positive domain-specific self-concept, and even the realistic evaluation of their academic achievements in line with their domain-specific self-concepts. This would mean that taken together under the umbrella term of “cognitive self-regulation” [4], enhanced EFs in terms of a bilingual advantage might be at the core of these children’s capability to execute realistic judgments. Under these assumptions, the bilingual advantage debate could be extended to posit a possible impact of superior EF on self-concept development in the frame of cognitive self-regulation.

## 7. Implications for Practice

The present study has substantial implications for practitioners and researchers working in any educational context. Positive self-concepts are essential for a healthy personality and identity development [10,64]. Hence, the importance of children’s self-concept enhancement and associated responsibilities of teachers, educational scientists, and psychologists seem to be obvious. The challenge lies in the individual promotion of all children including disadvantaged groups, as this provides a learning setting from which each child profits the most [65]. The present study has advanced our understanding of multilingual children’s academic self-concepts by reporting that monolinguals and multilinguals do not necessarily differ in the development of self-concepts. Enhancing children’s self-concepts and providing individual support for every child should be a vital aim for teachers, psychologists, and other practitioners. Crucially, this should start from the beginning of primary school as self-concepts already seem to decline by first-grade [13], and “early experiences of failure in learning to read have a lasting impact on a child’s self-beliefs, resulting in the emergence of a weak reader self-concept which tends to persist” [66] (p. 92).

The schools where the children in our study were attending offered great learning support on a teacher–student relationship level. These schools set an example for diversity management and equal treatment of children with and without migration background. Still, our results also revealed a difference in spelling performance in German, and it was found in conjunction with smaller lexicon size in German for the multilingual children of our sample [47]. We therefore suggest that more training and development of lexical skills are indispensable to help close the gap between the two groups also in terms of lexicon size and consequently, spelling in German.

## 8. Limitations and Directions for Future Research

In our study, we explored the connection between self-concepts and academic achievement using correlational analyses. As we did not investigate the direction of this relationship, we cannot make any statement concerning the causal ordering of the self-concepts and academic achievement [20,24]. Our findings reveal no differences between monolingual and multilingual children in academic self-concepts that imply disadvantages for multilingual children. The generalizability of these results may be further tested with a greater sample including a larger proportion of multilinguals allowing for subgroup comparisons. Such comparisons are highly interesting given that differences between the specific languages of the children occur [17], likely influencing children’s verbal performance in German and associated self-evaluations. Moreover, in the present study, we did not include the calculation of EF and self-concept, but rather have argued a theoretical link between them. These constructs merit testing in future studies. Finally, impending research could be dedicated to a more detailed assessment of different aspects of self-concepts; we focused on two facets of verbal self-concepts, which are, however, not representative of other domains such as mathematical skills.

## Figures and Tables

**Table 1:** Mean ranks per group (P: mono- and multilingual parents; C: mono- and multilingual third-graders) for parental aspirations towards educational achievements (e.g., spelling) and children’s willingness to learn (e.g., German language) (P, upper part) and for children’s evaluations of their teachers’ behavior towards them (C, lower part) based on questionnaire responses by parents or children.

Parents’ Aspirations (P) and Child Evaluations (C) of Their Teachers’ Behavior	Monolinguals Mean Rank	Multilinguals Mean Rank	Mann–Whitney Test
P: child should write text without errors	56.66	60.76	*U* = 1547 *p* = 0.441
P: child should make an effort in the German lessons	56.30	62.25	*U* = 1526 *p* = 0.281
C: my teachers like me	60.02	59.97	*U* = 1747 *p* = 0.993
C: my teachers treat me with fairness	58.58	60.67	*U* = 1655 *p* = 0.718
C: my teachers tell me off too often	59.05	58.94	*U* = 1693 *p* = 0.986
C: my teachers talk to me in a friendly way	59.02	57.87	*U* = 1631 *p* = 0.840
C: my teachers take care of me	57.38	62.09	*U* = 1585 *p* = 0.429

**Table 2:** Mean ranks for mono- and multilingual third-graders for domain-specific self-concepts.

Domain-Specific Self-Concepts	Monolinguals Mean Rank	Multilinguals Mean Rank	Mann–Whitney Test
Reading	63.31	55.88	*U* = 1530 *p* = 0.239
Spelling	65.25	53.46	*U* = 1402 *p* = 0.063

**Table 3:** Mean ranks for monolinguals and multilinguals for standardized tests of reading (reading comprehension–ELFE 1–6, reading fluency SLRT-II) and spelling (BUEGA) competences.

Standardized Test for Reading and Spelling	Monolinguals Mean Rank	Multilinguals Mean Rank	Mann–Whitney Test
Reading comprehension (n correct responses)	64.14	54.85	*U* = 1476 *p* = 0.144
Reading fluency (n correct responses)	58.86	60.32	*U* = 1673 *p* = 0.818
Spelling (n errors)	54.24	67.17	*U* = 1369 *p* = 0.042

**Table 4:** Correlations between self-concept for reading and standardized reading tests for monolinguals and multilinguals. Bivariate correlations between variables utilized Spearman’s rho.

		Reading Comprehension Test		Reading Fluency Test	
		Monolinguals	Multilinguals	Monolinguals	Multilinguals
Self-concept reading	*rₛ*	0.307	0.296	0.456	0.394
	*p*	0.012	0.031	<0.001	0.004

**Table 5:** Correlations between self-concept for spelling and the standardized spelling test for monolinguals and multilinguals. Bivariate correlations between variables utilized Spearman’s rho.

		Spelling Test	
		Monolinguals	Multilinguals
Self-concept spelling	*rₛ*	−0.392	−0.438
	*p*	0.001	0.001
